# Unilateral Headache Status after Intra-Aortic Balloon Pump Placement

**DOI:** 10.1155/2017/5043471

**Published:** 2017-07-27

**Authors:** Garret M. Weber, Alan L. Gass, Shalvi B. Parikh

**Affiliations:** ^1^Department of Anesthesiology, Westchester Medical Center, Valhalla, NY 10595, USA; ^2^Cardiac Transplantation and Mechanical Circulatory Support, Westchester Medical Center, Valhalla, NY 10595, USA

## Abstract

**Introduction:**

Intra-aortic balloon pump (IABP) counterpulsation is a catheter-based treatment for coronary artery disease and decompensated heart failure to increase coronary blood flow and improve cardiac output. IABP is generally well tolerated, and complications are usually related to peripheral vasculature or red blood cell and platelet consumption. The usual insertion site via femoral artery renders the patient bedbound. Recently, axillary artery has been used in patients with atherosclerotic peripheral vascular disease and documented small arteries or in those awaiting transplant to ensure ambulation and prevent deconditioning.

**Case Report:**

We present a patient with ischemic cardiomyopathy and severe left ventricular dysfunction, awaiting Orthotropic Heart Transplant. His worsening intractable angina and dyspnea necessitated IABP placement via left axillary artery, significantly improving his condition. He subsequently experienced migraine-type persistent unilateral headache refractory to standard pain management. Multiple strategies were utilized to treat his pain, but the patient insisted that his pain commenced after IABP placement. Ultimately, the removal of the pump led to complete resolution with no recurrence.

**Conclusion:**

The authors hypothesize that the unilaterally directed blood flow and direct increase in cerebral perfusion from the intra-aortic balloon pump may have caused vasodilation of the extracranial arteries, leading to a persistent and debilitating headache in this susceptible patient.

## 1. Introduction

Intra-aortic balloon pump (IABP) is a cylindrical polyethylene device inserted into the descending thoracic aorta, which increases myocardial oxygen delivery and cardiac output [1]. Its “counterpulsation” action causes inflation in diastole, which increases coronary perfusion via retrograde flow, while deflation during systole reduces afterload and increases forward blood flow [2]. The increase in myocardial oxygen supply and decrease in oxygen demand are utilized in ischemic myocardial states such as unstable angina and infarction, ventricular dysfunction with poor ejection fraction (EF), and cardiogenic shock [3]. Known complications include obstruction and compartment syndrome, distal ischemic events, aortic rupture, and thromboembolism. We report a patient with persistent unilateral headache following axillary IABP placement, hypothesized to be secondary to increased cerebral blood flow (CBF), with complete resolution of symptoms following removal of the pump.

## 2. Case Report

A 63-year-old male with ischemic cardiomyopathy and severe left ventricular dysfunction was admitted with chest pain and dyspnea at rest. He was on maximal medical therapy and had received multiple prior percutaneous coronary interventions and two previous coronary artery bypass surgeries. Recent coronary angiography revealed no possibility of further revascularization. He was evaluated for Orthotropic Heart Transplant (OHT) and listed as Status 1A-e (urgent need). His worsening intractable angina and dyspnea necessitated IABP placement via left axillary artery, which significantly improved his condition. Axillary artery was the chosen site of insertion (as opposed to femoral artery) in order to enable ambulation and prevent deconditioning, as the patient was awaiting transplant [4]. The IABP was placed under fluoroscopic guidance, with the tip of the IABP placed few centimeters below the aortic arch, as confirmed radiologically [5]. Following the procedure, the patient complained of new-onset, persistent, throbbing, and burning left-sided frontoparietal pain above the left lateral eyebrow that radiated to the left orbit, with an intensity of 7/10 on the numerical pain scale. It was aggravated by movement and relieved by pressure, with frequent watery eyes without visual changes. He had a remote history of traumatic brain injury due to motor vehicle accident but he denied any subsequent headache.

Examination revealed tender and bulging left temporal artery suspicious of temporal arteritis (TA); however, his normal erythrocyte sedimentation rate minimized TA as a likely diagnosis. Nonetheless, he was offered prophylactic prednisone, which he refused. Pain management service was consulted and multimodal treatment including acetaminophen, oxycodone, citalopram, and tizanidine was initiated, while nitrates were withheld [6]. The patient reported limited relief and continued to complain of a persistent pain in the same distribution. Subsequently, left supraorbital neuralgia was hypothesized, and gabapentin was initiated with limited benefit. A supraorbital block was offered, which was refused, and patient insisted on discontinuing the IABP.

In accordance with patient's wishes, the IABP was weaned and removed, and nitrates were initiated for angina prophylaxis. On postremoval assessment, the patient's headache had completely resolved with no future recurrence. He subsequently received OHT and was discharged with normal EF and no further headaches.

## 3. Discussion

Intra-aortic balloon pump (IABP) is a widely used circulatory assist device to improve coronary perfusion in acute coronary syndromes and improve cardiac output in acute decompensated heart failure. Coronary perfusion has often been compared to cerebral perfusion, drawing similarities in the pathophysiology and pharmacology of myocardial infarction and acute stroke [7, 8]. Augmentation of myocardial perfusion by epinephrine in cardiopulmonary resuscitation also enhances cerebral perfusion by reversing arterial collapse and eliminating systolic gradient between aortic and carotid pressures, thereby increasing cerebral perfusion pressure and flow [9].

One study showed that IABP augmentation causes a significantly higher increase in transcranial Doppler CBF velocity change in the middle cerebral artery with enhanced CBF especially in patients with preexisting left ventricular dysfunction [10]. Data indicates that IABP modifies the phasic profile of CBF to reflect the arterial pressure waveform due to counterpulsation [7]. There may also be a further increase in antegrade mean CBF velocity, especially in patients with intact cerebral autoregulation [11, 12]. Other studies suggest the use of IABP to optimize cardiac performance and maintain cerebral perfusion in symptomatic cerebral vasospasm following aneurysmal subarachnoid hemorrhage and neurogenic stress cardiomyopathy [13–16].

Further case reports suggest that IABP placement can increase proximal pressures and carotid blood flow above the IABP and decrease distal pressures and perfusion below it, leading to distal perfusion injuries (ischemia) [17, 18]. The authors of this case surmise that this effect may have caused increased CBF and headache in this patient after IABP placement. Anatomically, the tip of the axillary IABP rests few centimeters distal to the aortic arch as it passes through the left subclavian artery and into the descending aorta. This close proximity to the left carotid artery, the authors hypothesize, may also incur increased pressure and volume displacement during inflation, consequently raising diastolic pressures in the external and internal carotid arteries. Vasodilation of the superficial temporal artery, a major branch of external carotid artery, is a common cause of migraine, which may be a cause of severe unilateral headache in the presented patient as evidenced by the dramatic resolution of symptoms following IABP removal [19].
